# A Theory for Energy-Optimized Operation of Self-Adaptive Vibration Energy Harvesting Systems with Passive Frequency Adjustment

**DOI:** 10.3390/mi10010044

**Published:** 2019-01-09

**Authors:** Mario Mösch, Gerhard Fischerauer

**Affiliations:** Chair of Measurement and Control Systems, Center of Energy Technology (ZET), Universität Bayreuth, Universitätsstraße 30, D-95447 Bayreuth, Germany; mario.moesch@uni-bayreuth.de

**Keywords:** vibration energy harvesting, frequency tuning, electromagnetic, microgenerator, self-adaptive

## Abstract

Self-adaptive vibration energy harvesting systems vary their resonance frequency automatically to better exploit changing environmental conditions. The energy required for the adjustment is taken from the energy storage of the harvester module. The energy gained by an adjustment step has to exceed the energy expended on it to justify the adjustment. A smart self-adaptive system takes this into account and operates in a manner that maximizes the energy output. This paper presents a theory for the optimal operation of a vibration energy harvester with a passive resonance-frequency adjustment mechanism (one that only requires energy for the adjustment steps proper, but not during the hold phases between the steps). Several vibration scenarios are considered to derive a general guideline. It is shown that there exist conditions under which a narrowing of the adjustment bandwidth improves the system characteristics. The theory is applied to a self-adaptive energy harvesting system based on electromagnetic transduction with narrowband resonators. It is demonstrated that the novel optimum mode of operation increases the energy output by a factor of 3.6.

## 1. Introduction

In an increasingly interconnected future, distributed and embedded measurement systems become more important. Wireless sensor networks (WSN) collect large amounts of data and are used for environmental [1,2], energy-saving [3], and other purposes [4]. The sensors are currently powered either via cables, which is not possible everywhere, or by batteries. The proliferation of sensor nodes will therefore lead to a rise in battery use, which is detrimental for environmental reasons.

A potential solution to this problem is energy harvesting, i.e., the conversion of free environmental energy to usable electrical energy. In the last decade, much research has been devoted to vibration energy harvesting, by which the mechanical energy associated with common vibrations caused by passing cars, walking persons, slamming doors, etc. is converted to electrical energy [5,6]. The energy is converted from the mechanical to the electrical domain by electromagnetic [7] or piezoelectric micromachines [8] or hybrid forms [9]. Vibration energy harvesters are spring-mass-damper systems with a resonance frequency $f_{r}$ that depends on the effective spring stiffness $k_{\mathrm{eff}}$ and the effective mass m. The harvester will only be excited by ambient vibration frequencies $f_{a}$ close to $f_{r}$, otherwise the harvested power decreases sharply [10]. As the ambient vibration is imposed by the environment, an optimum match between $f_{a}$ and $f_{r}$ can only be achieved by using proper harvester designs. When $f_{a}$ varies with time, one can either adjust $f_{r}$ such that it tracks $f_{a}$ [10] or use a harvester with a broad enough resonance bandwidth such that $f_{a}$ remains within this bandwidth [9,10,11,12]. Here, we are concerned with the former case.

When a cantilever is used as a mechanical resonator in a vibration energy harvester, its resonance frequency can be adjusted by changing its clamping length [13], shifting the center of gravity of the mass [14], or changing the effective spring stiffness $k_{\mathrm{eff}}$. To implement the latter method, one can apply an additional load by, e.g., a voltage-controlled piezoelectric actuator [15,16,17,18,19] or by the force between two magnets, with one magnet attached to the cantilever and a tuning magnet at a variable distance nearby [20,21,22,23,24,25]. Smart materials, such as magnetorheological elastomers [26] and magnetostrictive materials [27], are used in the field, too.

A harvester is called self-adaptive if it detects a variation in the vibration frequency $f_{a}$ automatically and adjusts its resonance frequency $f_{r}$ on its own to maximize the harvested power. In principle, this constitutes a closed-loop control problem with $f_{r}$ as controlled variable and $f_{a}$ as reference variable. The final controlling element is the variable-distance dual-magnet system, actuated by a motor, [24,25] or the piezoelectric actuator [19] or whatever else may be used to influence $f_{r}$. The final controlled variable of the problem is the power output $P_{0}$ of the harvester. We use $f_{r}$ as intermediate controlled variable because there exists no reference variable for $P_{0}$ (we only know that we want to maximize it), whereas $f_{a}$ serves well as the reference variable for $f_{r}$, and because $P_{0}$ will be maximum for $f_{a}\approx f_{r}$. This introduces an important additional requirement not encountered in conventional control systems. The energy consumed by an adjustment step, for example the displacement of a tuning magnet against the magnetic force, is lost at the harvester output. An adjustment step only makes sense when the energy gained by it exceeds the energy expended on it. The control problem can then be stated as follows: make the resonance frequency $f_{r}$ follow the ambient vibration frequency $f_{a}$ unless the energy needed for this adjustment exceeds the energy gained by it.

It has been shown by Roundy and Zhang that a continuous adaptation process cannot meet the additional requirement; the energy required by the continuous adjustment is always greater than the energy gained by it [28]. What has not been investigated to the best of our knowledge is whether other modes of operation, such as discontinuous adaptation processes, behave differently.

This paper identifies the conditions for an advantageous operation of self-adaptive vibration energy harvesting systems. The work considers systems characterized by a high Q-factor, i.e., a narrow useful bandwidth, which are excited by a dominant sinusoidal vibration the frequency of which, however, periodically jumps. The assumption of essentially single-frequency excitation is realistic in many cases [29,30], and the assumption of frequency jumps follows the reasoning in [25]. This work is applicable to systems at the nano- to macro scale and of various geometries if the changing mechanical and electrical parameters as well as the harvested power are considered [7,31].

The paper is organized as follows. In Section 2, different adjustment modes and their applicability are discussed. In Section 3, important rules for single adjustment steps and periodic adjustment are derived. Section 4 presents methods to improve the net available power. In Section 5, the efficiency of the methods is demonstrated by way of an example. Section 6 serves to summarize the results.

## 2. Adjustment Modes

A self-adaptive resonant energy harvester is in one of two states at any given time. In the adaptation state, the resonator frequency is adjusted to the excitation frequency to maximize the power output; in the hold state, the resonator frequency is kept constant and the system harvests energy. The time spent in the adaptation state is very small compared to that spent in the hold state and therefore will be ignored. The adaptation and hold states alternate with each other. The state changes are triggered by external events, viz., by changes in the ambient vibration frequency $f_{a}$. In a way, the harvester performs some sort of frequency hopping controlled by the external excitation. Frequency hopping is used in spread spectrum modulation systems to avoid narrowband jammers, whereas the hopping process of the energy harvester serves to track a time-variant narrowband vibration. After each change of $f_{a}$, it must be decided if an adjustment step is required to increase the net power output of the harvester (‘net’ meaning output power minus power lost by the adjustment).

Switching the frequency in an energy harvester always consumes energy. One can distinguish three types of systems according to their behavior in the time intervals between the switching actions: active, semi-active, and passive [28]. Passive adjustment only requires energy during the adjustment step proper, but not in the hold state. This is exemplified by an energy harvester with a tuning magnet [22,23,25]. Energy is needed to move the magnet closer to or further away from another magnet on the mechanical resonator (e.g., a cantilever); however, no additional energy is required to keep the tuning magnet at a constant position owing to a locking mechanism. Note that the term “passive” does not mean an adapting system totally without the need of adjustment energy. The term differentiates between the energy requirements during the hold state, while the adjustment itself requires energy every time it is performed.

Semi-active adjustment works in a similar way to the passive adjustment, the only difference being the need for small amounts of energy from time to time in the hold state. By way of an example, Eichhorn et al. [19] change the effective spring constant $k_{\mathrm{eff}}$ with a piezoelectric actuator, the voltage of which has to be renewed every few minutes because of charge losses.

Finally, active adjustment continuously takes up energy in the hold state. Roundy [28] concludes that the energy dissipation of active adjustment exceeds the surplus energy generated by it. His reasoning is that the higher the vibration power, the higher the adjustment energy per time interval, a point that Zhu [10] contradicts. Zhu argues that the adjustment energy only depends on the frequency spacing ($f_{r}$ after the adjustment step minus $f_{r}$ before the step) and not on the excitation. However, even if an actively adjusted energy harvester effected an energy gain, it would still be inferior to a passively adjusted harvester in low-vibration environments because its adaptation mechanism consumes more energy. We therefore only consider passive adjustment in the rest of this paper, investigate its applicability, and derive a guideline for its optimum operation.

## 3. Derivation of Design Rules

### 3.1. Single Adjustment Steps

Let us assume that the vibration frequency $f_{a}$ changes and then remains constant for a time span τ equal to the duration of the next hold phase of the adaptation mechanism. One may think, e.g., of gearboxes or motors with variable revolution speeds [32]. After a single adjustment step from $f_{r,1}$ to $f_{r,2}=f_{a}$, the system harvests the energy $W_{0}=P_{0}\tau$, where $P_{0}$ denotes the average harvested power (all powers used in this work are time-averaged, or active, powers). The energy cost for the adjustment step is $W_{T}$, which is assumed to depend on the frequency spacing $\Delta f=|f_{r,2}-f_{r,1}|$ [10,25]. One method of varying $f_{r,1}$ is by changing a tuning-magnet gap, as $f_{r}$ depends on the gap width [20,21,22].

Introducing the frequency-dependent energy density (energy per frequency interval) ${\tilde{W}}_{T}(f)$, we may write the energy expended on the single adjustment step as [24,25]

(1) $$W_{T}=\int_{f_{r,1}}^{f_{r,2}}{\tilde{W}}_{T}(f)df.$$

The harvested energy $W_{0}$ is required to exceed $W_{T}$ to justify the adaptation:

(2) $$W_{0}=P_{0}\tau \overset{!}{\geq }\int_{f_{r,1}}^{f_{r,2}}{\tilde{W}}_{T}(f)\;df.$$

Whereas the adjustment energy $W_{T}$ can be estimated if one knows the function ${\tilde{W}}_{T}(f)$, the prediction of the possible harvested energy $W_{0}$ is much more difficult. One needs an estimate of both the expected average harvested power $P_{0}$ and the hold phase duration τ. This is often unfeasible in practice. For repeated adjustment steps, the best way would be to check that Equation (2) is fulfilled for every single step. When the required estimate is not available, a different methodology is called for.

One notices that the longer $f_{a}$ remains constant and the smaller Δf and ${\tilde{W}}_{T}$ are, the higher is the energy gain $W_{0}-W_{T}$. Obviously, a large frequency change followed by a short harvest time span is worse than a small frequency change followed by a long harvest time span. This is to be expected by common sense.

Ideally, the excess power $(W_{0}-W_{T})/\tau$ should exceed or at least equal the power $P_{L}$ delivered to a load. However, even adaptations resulting in smaller power gains could be beneficial if they were to reduce the amount of energy drawn from the energy storage.

### 3.2. Periodic Adjustment

A time-varying vibration frequency $f_{a}$ requires repeated adjustment. In practice, a change in $f_{a}$ could occur at any time. The time instants of the frequency changes need not be equidistant, and they need not follow a deterministic law. This means that $f_{a}$ is a stochastic process in general ([33], p. 825). To keep our treatment simple, we follow [25] in that we assume that the frequency $f_{a}$ of the single-frequency vibration varies randomly within a frequency band from $f_{a,\mathrm{low}}$ to $f_{a,\mathrm{high}}$, but that the frequency jumps occur at discrete instants of time separated by equidistant intervals τ. This simplified model describes the processes well on average, if τ is chosen to be equal to the expected value of the time span between two successive vibration frequency jumps. In a sense, we have replaced a non-equidistantly sampled signal by a signal with periodic sampling. This then calls for periodic adjustment steps. The system adapts from $f_{a,\mathrm{old}}$ to $f_{a,\mathrm{new}}$ whenever a frequency jump occurs. The harvested power $P_{0}$ is considered to be independent of the frequency because the harvester is always operated at its optimum working point.

The average frequency spacing is $\bar{\Delta f}$, and the adjustment energy required per frequency interval, ${\tilde{W}}_{T}$, is modeled as a frequency-independent constant as in [19,23]. The latter detail amounts to the assumption that the energy expended on a single adjustment step is merely a function of the frequency difference before and after the adjustment (and not, e.g., of the absolute frequency before the adjustment). This then leads to an average adjustment energy of

(3) $$W_{T}={\tilde{W}}_{T}\bar{\Delta f}$$

and to an average adjustment power of $P_{T}=W_{T}/\tau$. Notice that we use energies for single adjustment steps, but powers for periodic adjustment as the energy of any infinitely periodic signal is infinite. The adjustment process is now consuming power with a constant $P_{T}$.

As the harvested power $P_{0}$ should exceed the adjustment power $P_{T}$, the net available power $P_{\mathrm{net}}$ has to meet the following condition:

(4) $$P_{\mathrm{net}}=P_{0}-P_{T}=P_{0}-\frac{W_{T}}{\tau }\overset{!}{>}0.$$

**Figure 1:**
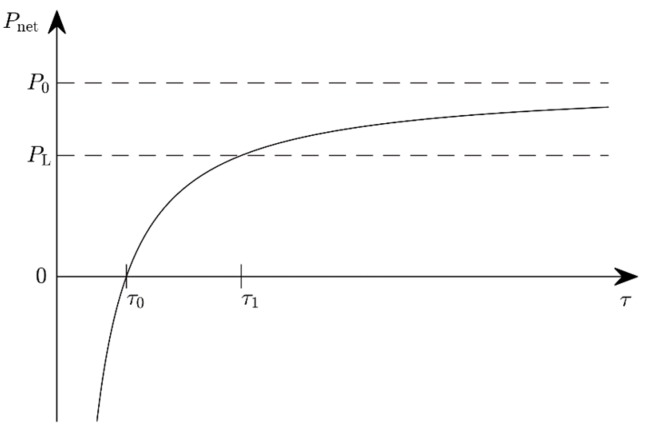
The available power *P*_net_ for periodic adjustment as a function of the time span τ between adjustment steps.

For sufficiently rare frequency changes and therefore sufficiently rare adjustment steps (τ large), $P_{\mathrm{net}}$ is positive as required (Figure 1). At $\tau_{0}=W_{T}/P_{0}$, the harvested and adjustment powers are equal and $P_{\mathrm{net}}=0$, so one could deactivate the adjustment altogether without a detrimental effect. $\tau <\tau_{0}$ is associated with a fast-changing environment so that the adjustment cost exceeds the benefit. The adjustment is to be avoided in this case. For $\tau \geq \tau_{1}=W_{T}/P_{0}-P_{L}$, $P_{\mathrm{net}}$ is big enough so that a load can be supplied with a power $P_{L}$. (Notice that a self-adaptive energy harvester needs an acceleration sensor or some other frequency-measuring element that constitutes an additional consumer.) Very rare changes of the ambient vibration frequency $f_{a}$ ($\tau \gg \tau_{1}$) render the adjustment power $P_{T}$ negligible and so $P_{\mathrm{net}}\approx P_{0}$.

This discussion explains why fast-changing environments are problematic for periodic adjustment. As one cannot increase the hold-phase duration τ imposed by the environment, one must resort to other methods of reducing the average adjustment power $P_{T}$. This is the subject of the next section.

## 4. Optimization of Net Available Power

### 4.1. Omission of Adjustment Steps

**Figure 2:**
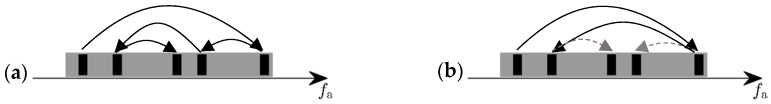
Omission of adjustment steps. The black bars mark a sequence of random ambient vibration frequencies, and the black and grey arrows respectively represent successive and omitted resonance frequency jumps of the energy harvester. (**a**) The harvester tracks the ambient resonance closely, and every possible adjustment step is performed (*r* = 1). (**b**) Every other step is omitted (*r* = 1/2).

An obvious method for reducing the energy loss caused by too-frequent adjustment is to skip adjustment steps selectively. We define the decimation ratio r as the number of adjustment steps in a (large) time interval divided by the number of steps that would have been possible if one had adjusted at every ambient vibration frequency jump. The decimation ratio is also the ratio between τ (the average time span between two successive jumps in $f_{a}$) and the average time span $\tau_{\mathrm{adj}}$ between two successive adjustment steps. It is a number between 0 (no adjustment at all) and 1 (adjustment whenever $f_{a}$ jumps). Figure 2 illustrates this for the example of r=1/2. Each of a sequence of adjustment steps has to satisfy Equation (2). The average difference $\bar{\Delta f}$ between the harvester resonance frequencies remains unchanged.

For a better understanding of the effects of periodic adjustment, consider the influence of the decimation ratio r on the average adjustment power with $P_{\mathrm{Tm}}=P_{T}(r=1)$ being the average tuning power when every adjustment step is performed (r=1):

(5) $$P_{T}(r)=P_{\mathrm{Tm}}\cdot r.$$

We assume that the harvester with a typical high Q-factor collects no energy at all when $f_{r}$ deviates from $f_{a}$. That this assumption is not a major source of errors can be shown as follows. The relative half-power bandwidth (HPBW) of a resonant system with Q-factor *Q* is 1/*Q* ([34], pp. 276–278). For systems with Q>200 [30], this amounts to a numerical value of less than 0.5%. A typical tuning range of a practical harvester is ±20% of its quiescent frequency (much less jeopardizes the advantages of the adaptivity, much more may be hard to achieve). Hence, for uniformly distributed ambient vibration frequencies, the probability that the vibration frequency jumps to a value within the HPBW of the harvester becomes 0.5%/40% = 0.0125. It does not make much of a difference for the total energy output of a harvester if one computes the energy harvested in these rare cases in detail and does not adapt or if one treats this energy as zero and adapts as we have assumed for simplicity’s sake. The harvested power $P_{0}$ then depends on the decimation ratio with the maximum harvested power $P_{0m}=P_{0}(r=1)$ as

(6) $$P_{0}(r)=P_{0m}\cdot r.$$

The omission of adjustment steps should provide an advantage over strictly periodic adjustment to justify the omission. This is equivalent to the requirement that

(7) $$(P_{0m}-P_{\mathrm{Tm}})r\overset{!}{>}P_{0m}-P_{\mathrm{Tm}}.$$

The inequality can only be satisfied for allowed values of *r* (between 0 and 1) when $P_{0m}<P_{\mathrm{Tm}}$. This describes a harvester that loses energy with each adjustment step. The decimation then affects the adjustment power and the harvested power in the same way, which reduces the energy loss. However, a self-adaptive harvester with an adjustment mechanism so costly that it consumes energy rather than providing extra energy cannot be turned into a more effective system by the omission of adjustment steps. The complete deactivation of the adaptation mechanism in such a harvester would avoid losses altogether (limit case r=0), and would outperform any other parameter settings meant to reduce the losses in the system.

The overall conclusion is that the omission of adjustment steps can never increase the energy output of a harvester.

### 4.2. Scaling of the Adjustment Bandwidth

Another method for reducing the energy loss caused by too-frequent adjustment is to reduce the adjustment bandwidth, defined as the span of the frequency interval covered by all resonance frequencies allowed in the next adjustment step. Such a reduction should shrink the average value of the spacing Δf between the harvester frequencies before and after adjustment and, consequently, the adjustment power.

#### 4.2.1. Upper Limit for the Frequency Spacing

**Figure 3:**
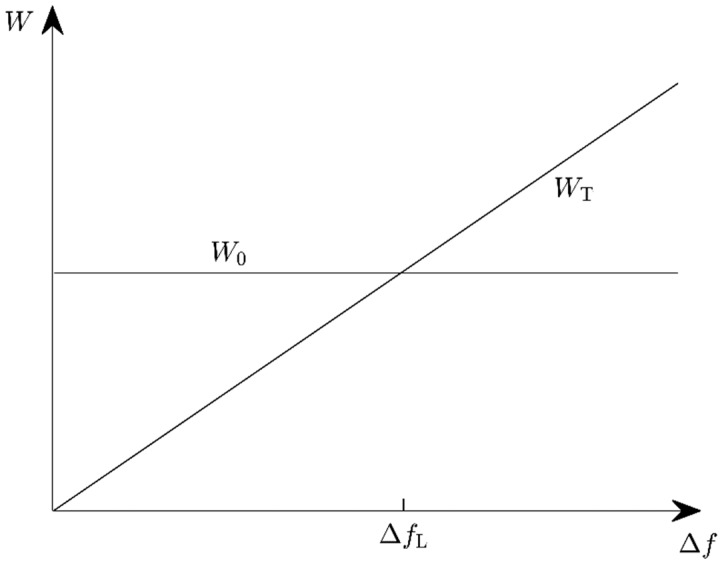
The possible harvested energy *W*_0_ and required adjustment energy *W*_T_ as functions of the frequency spacing Δ*f* between the harvester resonance frequencies before and after adjustment.

**Figure 4:**
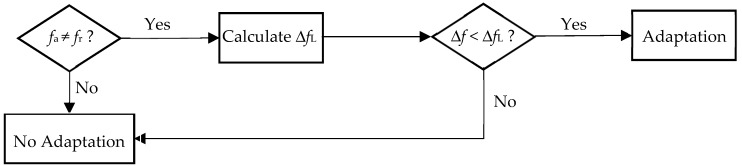
The decision algorithm for a single adaptation step.

Let us assume, as before, that the energy expended to switch the resonance frequency by Δf depends only on Δf, but not on the instantaneous resonance frequency $f_{r}$ prior to the adjustment. The adjustment energy per step, $W_{T}$, is again given by Equation (3) with frequency-independent ${\tilde{W}}_{T}$. The energy harvested during the following harvest phase is $W_{0}=P_{0}\tau$, independent of frequency, when the adjustment step is performed ($f_{r}=f_{a}$), and $W_{0}=0$ otherwise. The harvester should be adjusted only if $W_{0}-W_{T}>0$, or

(8) $$\Delta f<\Delta f_{L}=\frac{P_{0}\tau }{{\tilde{W}}_{T}}$$

with the upper limit $\Delta f_{L}$ of the frequency spacing (Figure 3). It must be decided before every adjustment step if the resulting frequency change Δf would remain below the limit value $\Delta f_{L}$ (Figure 4).

It is obvious that a large frequency change in an adjustment step incurs a high energy cost. Depending on the vibration force and the system properties, such an adjustment is detrimental because the harvested energy is unable to make up for the adjustment energy. As mentioned in Section 3.1, the practical use of this insight is difficult as estimates for the average harvested power $P_{0}$ and the average time span τ between successive frequency jumps of the ambient vibration in Equation (8) may not be readily available. However, it can be stated in general that a reduced average frequency spacing $\bar{\Delta f}$ increases the available power $P_{\mathrm{net}}$ for periodic adjustment.

This is all the more true in reality, as many energy harvesting systems employ an adaptation technique that applies an additional mechanical load to the structure [19,24,25]. The higher this load, the higher the mechanical damping and the lower the possible harvested power $P_{0}$. $P_{0}$ then is a function of the frequency, which could be modeled as $x(f_{r})P_{0}$ with $x(f_{r})\leq 1$. We have made no attempt to include such a frequency-dependent damping influence in our adaptation algorithms. Taking it into account would lead to a smaller value of the upper limit $\Delta f_{L}$ of the frequency spacing. The effect is less severe when only small adjustment steps around the quiescent resonance frequency are performed.

#### 4.2.2. Rules for a Periodic-Adjustment System

**Figure 5:**
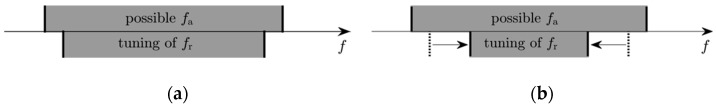
Two adjustment-range strategies. (**a**) State A. (**b**) State B, with a narrowed adjustment bandwidth relative to state A (*s* < 1).

To follow up on this idea, we consider a periodic-adjustment system as described in Section 3.2. The system is assumed to be in a state A, in which the adjustment bandwidth is smaller than or equal to the maximum bandwidth, the latter being identical to the frequency span containing all possible frequencies $f_{a}$ (Figure 5a). The adjustment bandwidth is then scaled by a factor of s, which brings the system into state B (Figure 5b). The process is called adjustment-bandwidth scaling (ABS) in the following. For s>1, the adjustment bandwidth is increased; for s<1, it is narrowed; and for s=1, it remains unchanged, which means that state B is identical to state A. The harvester only adjusts its resonance frequency $f_{r}$ to $f_{a}$ when $f_{a}$ is inside the new adjustment bandwidth, otherwise the adjustment step is omitted (resulting in $W_{T}=0$ and $W_{0}=0$). In the following, an index A or B indicates that the associated variables respectively correspond to state A and state B.

The average harvested power after ABS is

(9) $$P_{0,B}(s)=P_{0,A}\cdot s.$$

The adjustment power is

(10) $$P_{T,B}(s)=P_{T,A}\cdot s^{2}$$

because it depends on the average spacing ${\bar{\Delta f}}_{B}(s)={\bar{\Delta f}}_{A}\cdot s$ between the harvester frequencies before and after adjustment (see Equation (3)). The net power in state B has to exceed the net power in state A to justify the scaling:

(11) $$P_{\mathrm{net},B}=P_{0,B}-P_{T,B}=P_{0,A}s-P_{T,A}s^{2}\overset{!}{>}P_{\mathrm{net},A}=P_{0,A}-P_{T,A}.$$

This then leads to a gain in the net available power of

(12) $$\Delta P_{\mathrm{net}}(s)=P_{\mathrm{net},B}-P_{\mathrm{net},A}=(s-ks^{2}-1+k)\cdot P_{0,A}\geq 0$$

with the power ratio $k=P_{T,A}/P_{0,A}$ introduced for brevity’s sake. Equation (12) may be considered as an inequality for the scaling factor *s*. The limiting case $\Delta P_{\mathrm{net}}(s)=0$ (the scaling is neither advantageous nor harmful) constitutes a second-order equation for *s*. This equation has two zeros: $s_{1}=1$ describes state A and $s_{2}=(1-k)/k$ depends on the power characteristics of state A. Every scaling factor lying between these zeros yields more available power than the system in state A. The optimum scaling is

(13) $$s_{\mathrm{opt}}=\frac{1}{2k}$$

leading to the optimum gain

(14) $$\Delta P_{\mathrm{net},\;\mathrm{opt}}=(k+\frac{1}{4k}-1)\cdot P_{0,A}.$$

**Figure 6:**
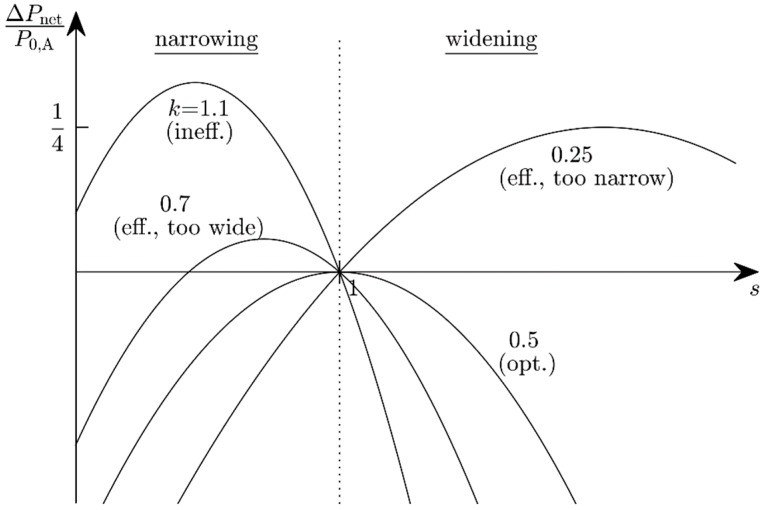
The change Δ*P*_net_ in net available power as a function of the adjustment-bandwidth scaling (ABS) factor *s* with the power ratio *k* (=adjustment power divided by power harvested prior to the ABS) as a parameter.

The fractional change in available power obviously is a function of the power ratio k (Figure 6) and the state-A efficiency:

- Efficient harvester, but too narrowband ($k<\frac{1}{2}$, $s_{2}>1$): An adjustment bandwidth reduction improves nothing, but a widening is advantageous for $1<s<s_{2}$. This is only possible when the adjustment bandwidth in state A is not the potential maximum.
- Optimum harvester ($k=\frac{1}{2}$, $s_{2}=s_{1}=1$): Limiting case, no change in the adjustment bandwidth can improve the system efficiency, because it is already at the potential maximum.
- Efficient harvester, but too wideband ($\frac{1}{2}<k<1$, $0<s_{2}<1$): Narrowing is advantageous for $s_{2}<s<1$, widening never.
- Inefficient harvester (k>1): $s_{2}$ would be negative, which is not admissible for physical reasons. Narrowing is always advantageous (0<s<1) because the harvester is in a state in which it expends more energy on its adaptivity than it gains from it.

When $P_{\mathrm{net},B}$ is negative, the bandwidth reduction is avoided and the adaptation mechanism is turned off altogether, even though $\Delta P_{\mathrm{net}}$ may be positive. The latter is a necessary, but not a sufficient condition. Consider, e.g., an inefficient state A with $P_{T,A}=5P_{0,A}$ and $P_{\mathrm{net},A}=-4P_{0,A}$. s=1/2 leads to an energy gain and $\Delta P_{\mathrm{net}}=3.25P_{0,A}$. However, $P_{\mathrm{net},B}=-0.75\;P_{0,A}$, so the system still loses energy. A sufficient condition for ABS therefore is $\Delta P_{\mathrm{net}}>0$ and $P_{\mathrm{net},B}>0$. This leads to the following restrictions for the ABS factor *s* in the various power-ratio regimes:

- $k<\frac{1}{2}$: $1<s<s_{2}$,
- $\frac{1}{2}<k<1$: $s_{2}<s<1$,
- k≥1: $s<\frac{1}{k}$.

**Figure 7:**
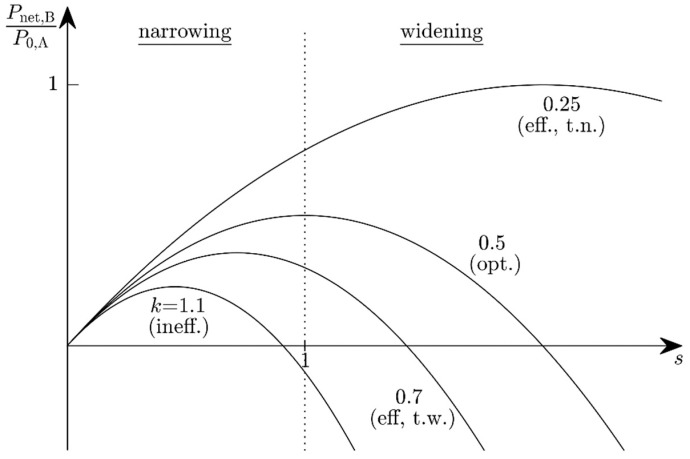
*P*_net,B_ after ABS with a factor of *s* with the power ratio *k* (=adjustment power divided by power harvested prior to the ABS) as a parameter.

**Figure 8:**
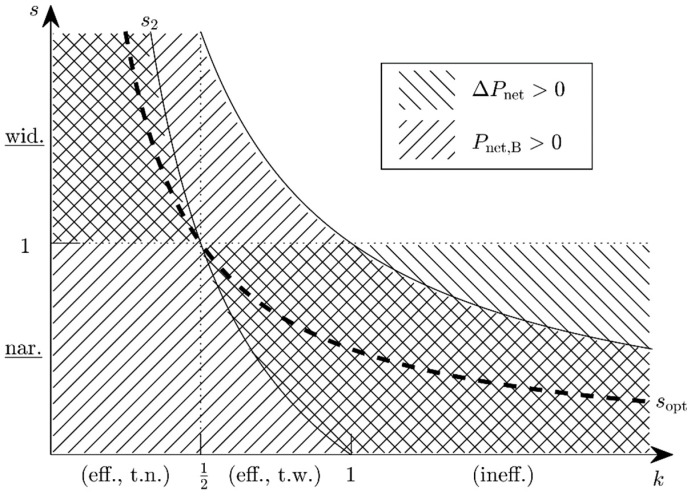
Regions in the *s*-*k*-domain for which the condition Δ*P*_net_ >0 is satisfied (hatched from northwest to southeast) and for which the condition *P*_net,B_ >0 is satisfied (hatched from southwest to northeast). The crosshatched region marks all parameter combinations associated with an energy harvester the energy output of which may be improved by ABS. The optimum performance is achieved for parameter combinations on the dashed line marked with *s*_opt_.

For the above example (k=5), this implies s<1/5. Figure 7 shows the available power $P_{\mathrm{net},B}$ as a function of the ABS factor *s* for various power ratios *k*. The optimum scaling factors are shown in Figure 8. Depending on the state-A power ratio, there exist scaling factors for which the available power will both improve and be positive. The optimum performance is reached with $s_{\mathrm{opt}}$.

**Figure 9:**
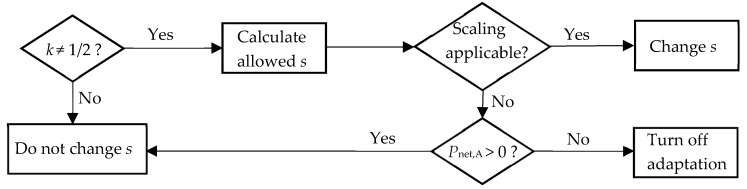
The decision algorithm for the scaling of the adjustment bandwidth.

The algorithm for such ABS is shown in Figure 9. For k≠1/2, the allowed scaling factors are calculated from $P_{0,A}$ and $P_{T,A}$. If the factors can be achieved physically, the adjustment bandwidth is scaled, preferably with $s_{\mathrm{opt}}$. Otherwise, one needs to decide whether the adaptation is turned off or not. For positive $P_{\mathrm{net},A}$ the adaptation is continued with unchanged s, otherwise it is turned off to save energy.

The load power $P_{L}$ is not taken into account for this decision. The load can be supplied with $P_{L}$ whenever $P_{\mathrm{net},B}\geq P_{L}$, which leads to:

(15) $$s^{1/2}=\frac{P_{0,A}\pm \sqrt{{P_{0,A}}^{2}-4P_{T,A}P_{L}}}{2P_{T,A}}$$

Real-valued zeros only exist for $P_{0,A}^{2}\geq 4P_{T,A}P_{L}$, otherwise a constant supply of $P_{L}$ is not possible even though the above scaling conditions might be met.

One also notices that a widening of the adjustment bandwidth (s>1) is only possible until the maximum potential bandwidth is reached. This maximum can either be the physical maximum of the adjustment bandwidth or the interval containing all possible values of $f_{a}$. If s were to cause a widening beyond the maximum, no scaling can improve the system efficiency.

## 5. Validation by Application to an Implemented System

### 5.1. System Description and Analysis

It was deemed best to demonstrate the usefulness of the design rules derived in Section 4.1 and Section 4.2 by applying them to an existing harvester. Any modification of such a harvester can influence its energy output, which would have interfered with our goal of exclusively evaluating the effectivity of algorithmic changes in its mode of operation. We therefore resorted to a self-adaptive harvester that is extremely well-documented in the literature [25], but did not replicate it to avoid inadvertent system changes. Instead, we applied our design rules to the documented system.

The system by Hoffmann et al. [25] comprises a cantilever resonator and vibration energy harvesting by Faraday’s law of induction. A magnet is mounted on the cantilever near its end. A stationary nearby tuning magnet is used to adjust the resonance frequency of the cantilever. This tuning magnet is cylindrical with a diametrical magnetic polarization. Depending on the rotation angle, which is changed in steps of 15° by a stepper motor, the cantilever magnet “sees” either the north or the south pole of the tuning magnet. This influences the magnetic force between the magnets and therefore the resonance frequency $f_{r}$ of the harvester owing to mechanical loading. At the smallest magnet distance, $f_{r}$ can be varied between 31 and 49 Hz. One 15° rotation requires 124 mJ of energy and changes $f_{r}$ by 2 Hz at most. A single adjustment step can consist of one or several 15° rotations. The system adjusts immediately whenever the vibration frequency $f_{a}$ changes, and the $f_{a}$ changes occur at equidistant time intervals. This amounts to a periodic adjustment scheme, the adjustment being performed by setting the rotation angle to be appropriate for the current $f_{a}$. The average frequency spacing is $\bar{\Delta f}=8\;\mathrm{Hz}$, which is equivalent to a relative adjustment bandwidth of (8 Hz)/(49 Hz−31 Hz)≈0.44. This is greater than the theoretical value of $\frac{1}{3}$ for equally distributed $f_{a}$ (see Appendix A). The physical system includes power management with a microcontroller and a capacitor as an energy storage unit (C=0.6 F). $f_{a}$ is measured by the time period between two zero crossings of the pickup coil voltage.

In [25], the energy harvester was experimentally characterized with a predetermined sequence of vibration frequencies involving eight different frequencies. In the following, an index *i* with *i* = 1, 2, …, 8 indicates that the associated variable refers to phase *i*. For instance, $f_{a,3}$ is the vibration frequency in phase 3, $W_{0,5}$ denotes the energy harvested in phase 5, and $W_{T,2}$ is the energy required to adjust from $f_{a,1}$ to $f_{a,2}$. The sequence of vibration frequencies $f_{a,i}$ was $f_{a,1}=40\;\mathrm{Hz}$, $f_{a,2}=35\;\mathrm{Hz}$, $f_{a,3}=47\;\mathrm{Hz}$, $f_{a,4}=38\;\mathrm{Hz}$, $f_{a,5}=45\;\mathrm{Hz}$, $f_{a,6}=49\;\mathrm{Hz}$, $f_{a,7}=40\;\mathrm{Hz}$, and $f_{a,8}=31\;\mathrm{Hz}$. Each frequency was applied for 70 s (hence, in our notation, τ=70 s). After the eighth frequency, the same sequence would start again with frequency no. 1. The acceleration amplitude was kept constant at $2\;{m/s}^{2}$.

**Figure 10:**
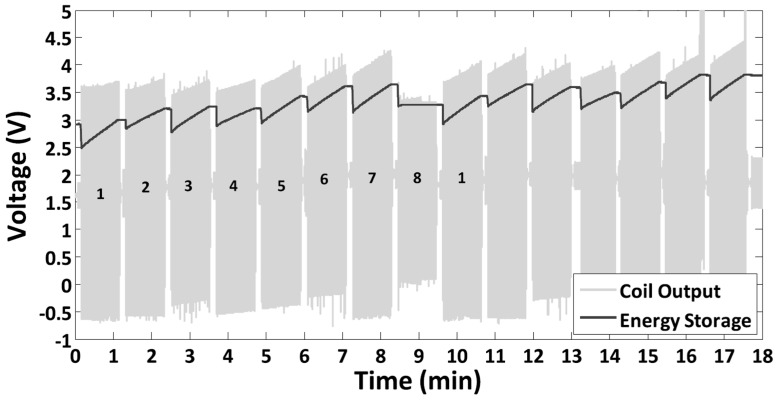
Voltage of the energy storage and coil output voltage of the physical system in Reference [25].

Hoffmann et al. [25] carried out an experiment without an electrical load. They present the capacitor voltage as a function of time (Figure 10). The frequency adaptation was repeated until the capacitor voltage $V_{C}$ reached 3.8 V, starting from 2.9 V. The reference was the same system without adaptation ($f_{r}=38\;\mathrm{Hz}$), in which case $V_{C}=3.8\;V$ was reached after 33 min (energy only harvested in time intervals with $f_{a}=38\;\mathrm{Hz}=f_{r}$). With the adaptation mechanism turned on, $V_{C}=3.8\;V$ was reached after 16.5 min. This was an impressive demonstration of the fact that the periodic adjustment of the harvester frequency to the ambient frequency can indeed increase the energy output.

Reference [25] reports a harvested power of $P_{0}=P_{\mathrm{net}}=0.9\;\mathrm{mW}$ for the non-adapting system and $P_{0}=8.5\;\mathrm{mW}$, $P_{T}=6.7\;\mathrm{mW}$, and $P_{\mathrm{net}}=1.8\;\mathrm{mW}$ for the periodically adapting system. These values are associated with the specific start and stop points chosen but do not correspond to averages valid for sequences periodically repeated infinitely many times. As can be seen in Figure 10*,* the second sequence was not completed, but was interrupted after hold phase 7. The omitted hold phase 8 would have yielded no power harvest ($P_{0}=0$ because of a too-low coil voltage), but the energies to be expended to adjust from $f_{a,7}$ to $f_{a,8}$ and back from $f_{a,8}$ to $f_{a,1}$ would have been lost. We have calculated the infinite-sequence net available power $P_{\mathrm{net}}$ from the difference of the capacitor voltages at the very start of two successive hold phases 1 and the time duration of a full sequence involving eight frequencies. This resulted in $P_{0}=0.85\;\mathrm{mW}$ for the non-adjusting system and $P_{0}=10.1\;\mathrm{mW}$, $P_{T}=8.7\;\mathrm{mW}$, and $P_{\mathrm{net}}=1.3\;\mathrm{mW}$ for periodically adjusting system. These were the base values for the following evaluation of the design-rule effectivity.

### 5.2. Results for a Fixed-Process Stationarity Time

Applying the rule for single tuning steps, Equation (2), we obtain the following results:

- Adjusting to $f_{a,8}$ should be avoided as $W_{T,\;8}>W_{0,\;8}=0$. This would also save the adjustment energy $W_{T,\;1}$ from $f_{a,8}$ to $f_{a,1}$ because $f_{a,7}=f_{a,1}$.
- Adjusting to $f_{a,4}$ should be avoided because the capacitor voltage decreases in hold phase 4, so $W_{T,\;4}>W_{0,\;4}$. Notice that, in contrast to hold phase 8, the energy $W_{0,\;4}$ harvested in hold phase 4 would have exceeded the adjustment energy $W_{T,\;4}$ if the hold phase duration τ had been longer.

**Figure 11:**
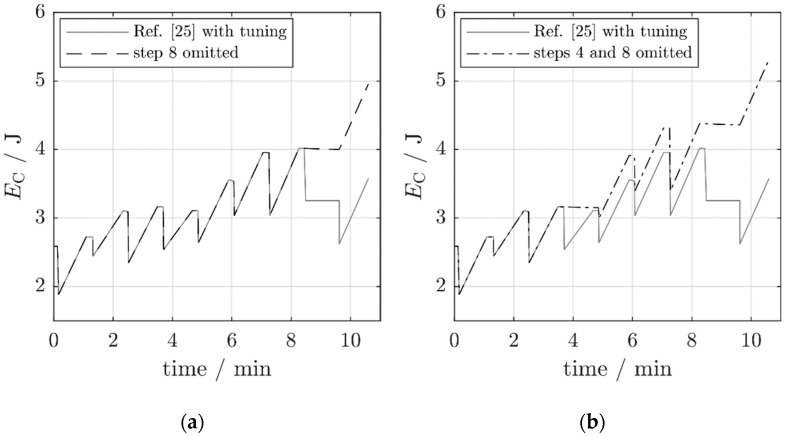
The capacitor energy for various operating modes of the self-adaptive harvester from Reference [25]. (**a**) Periodic adjustment with step 8 omitted. (**b**) Periodic adjustment with steps 4 and 8 omitted.

The results of these measures are visualized in Figure 11. The chart presents the capacitor energy as a function of time during the first eight-frequency sequence of Figure 10 (the capacitor voltage of Figure 10 was transformed to an energy curve by the relation $E_{C}=\frac{1}{2}\;CV_{C}^{2}$). The improvement over the strictly periodic adjustment strikes the eye. The main contribution is due to the omission of the adjustment from $f_{a,7}$ to $f_{a,8}$ and from $f_{a,8}$ to $f_{a,1}$ (an energy increase of 0.94 J; Figure 11a). The omission of the adjustment to $f_{a,4}$ contributes another 0.36 J (Figure 11b). The net available power $P_{\mathrm{net}}$ in the two improved scenarios increases from 1.3 mW to respectively 3.8 mW (by a factor of 2.9) and 4.4 mW (by a factor of 3.4). This is not the result of an increase in the harvested power $P_{0}$—it even decreases because of the missing harvest during hold phase 4—, but of a substantial decrease in the tuning power $P_{T}$.

A further improvement is achieved with ABS. By Section 4.2.2, the optimum scaling factor is $s_{\mathrm{opt}}=P_{0,A}/\left(2P_{T,A}\right)=10.1\;\mathrm{mW}/(2\cdot 8.7\;\mathrm{mW})\approx 0.58$. The prior adjustment frequency range of 40±9 Hz is reduced to 40±0.58⋅9 Hz. It shows that the only vibration frequencies within this range (and to which the harvester should now be tuned) are $f_{a,1}$, $f_{a,2}$, $f_{a,4}$, $f_{a,5}$, and $f_{a,7}$. (Note that this only holds for τ=70 s. Other values of the process stationarity time, i.e., the time span during which the process does not change its characteristics, would have involved other values of $P_{0}$ and $P_{T}$ and, therefore, a changed $s_{\mathrm{opt}}$.)

**Figure 12:**
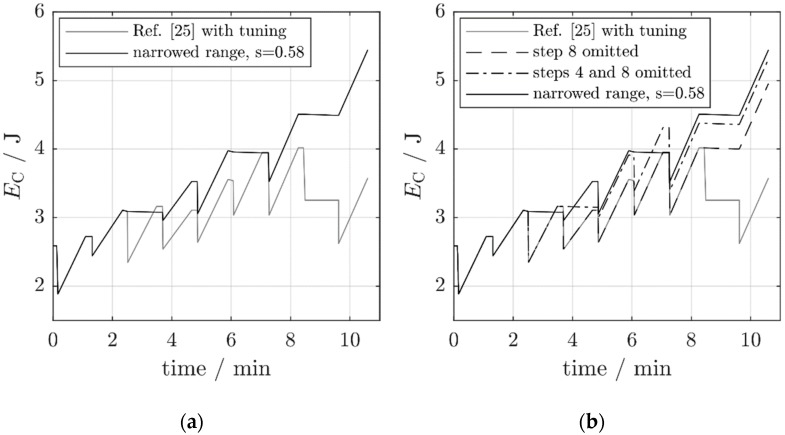
The capacitor energy for various operating modes of the self-adaptive harvester from Reference [25]. (**a**) Operation with a narrowed adjustment bandwidth (s = 0.58). (**b**) Comparison with the results from Figure 11.

The average powers after the scaling are $P_{0,B}=6.9\;\mathrm{mW}$, $P_{T,B}=2.3\;\mathrm{mW}$, and $P_{\mathrm{net},B}=4.7\;\mathrm{mW}$. The latter value is an improvement by a factor of 3.6 over the strictly periodic adjustment without a hopping-bandwidth reduction. Even the adjustment to $f_{a,4}$ is advantageous because the omission of the adjustment to $f_{a,3}$ reduces the adjustment energy to $f_{a,4}$ (now from $f_{a,2}$). Overall, the average adjustment energies have become smaller because the average frequency spacing $\bar{\Delta f}$ has decreased. As demonstrated by the charts in Figure 12, the narrowing of the adjustment bandwidth even outperforms the single-step rules.

### 5.3. Influence of the Process Stationarity Time

**Figure 13:**
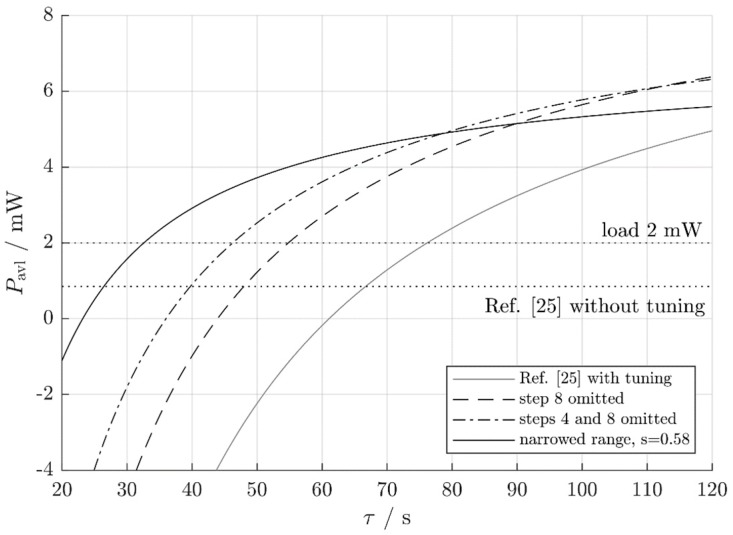
The available power for various operating modes of the self-adaptive harvester from Reference [25].

Hoffmann et al. [25] also attempted to supply a constant load with $P_{L}=2\;\mathrm{mW}$. Both the non-adapting system ($P_{\mathrm{net}}=0.85\;\mathrm{mW}$) and the periodically adapting system ($P_{\mathrm{net}}=1.3\;\mathrm{mW}$) missed this target. This result, however, depends on the time separation τ between vibration frequency changes (the process stationarity time). The previous results were based on a value of τ=70 s. With τ=80 s, even the non-optimized periodically adjusting system would be able to supply its load permanently with a power of 2 mW. This is because longer hold phases τ reduce the average adjustment power $P_{T}$, whereas $P_{0}$ stays constant (see Equation (4)). The improved tuning modes of the present work result in specific lower limits $\tau_{0}$ and $\tau_{1}$ above which the net available power is respectively positive and greater than 2 mW (Figure 13 and Table 1). Each of the improved operating modes leads to smaller $\tau_{0}$ and $\tau_{1}$ or, to put it differently, it can cope with faster varying environments than the original harvester. Table 1 lists the results obtained with the various modes of operation, again demonstrating the fact that a given harvester can deliver additional energy if operated appropriately.

It depends on τ which adjustment mode yields the best results. Very frequent frequency changes (small τ) would result in a high $P_{T}$, so the non-adjusting system outperforms any adjusting system (Reference [25] without adjustment in Figure 13). However, the 2-mW load power cannot be supplied permanently in this case. For slightly less frequent changes, the narrowing of the adjustment bandwidth is the optimum method. The adjustment is still expensive, and the most expensive adjustment steps should be skipped. For even less-frequent changes, omitting the adjustment steps 4 and 8 or omitting step 8 alone lead to the highest net energy harvest, depending on the results of hold phase 4. For infrequent changes (very high τ), a strictly periodic adjustment as in Reference [25] outperforms the adjustment with a narrowed bandwidth because $P_{T}$ decreases with τ.

The scaling factor s=0.58 is the optimum value for τ=70 s. Other values of τ lead to other optimum scaling factors $s_{\mathrm{opt}}$. The ABS factor s is a function of the hold phase duration τ because this duration affects the adjustment power $P_{T,A}$ and, consequently, the power ratio $k=P_{T,A}/P_{0,A}$. The harvester from Reference [25] is characterized by $P_{T,A}=8.7\;\mathrm{mW}\times 70\;s/\tau$ and k≈60 s/τ (see Table 1). By Section 4.2.2, the advantageous and the optimum scaling factors in the different power-ratio regimes (expressed by the associated process stationarity times τ) are:

- τ<48 s: the load cannot be supplied with $P_{L}=2\;\mathrm{mW}$ continuously (Equation (15)).
- 48 s<τ<60 s: narrowing the adjustment bandwidth always pays off, and $s_{\mathrm{opt}}=120\;s/\tau$.
- 60 s<τ<120 s: a narrowing pays off for 1<s<60 s/(τ−60 s), and $s_{\mathrm{opt}}=120\;s/\tau$.
- τ≈120 s: ABS does not improve the system.
- τ>120 s: widening the adjustment bandwidth is advantageous for 1>s<60 s/(τ−60 s), and $s_{\mathrm{opt}}=120\;s/\tau$.

### 5.4. Influence of a Frequency Dependence of the Adjustment Power

In the preceding Sections, the adjustment energy per frequency interval, ${\tilde{W}}_{T}(f)$, was assumed to be a frequency-independent constant (see Equation (3)). This need not be the case. In fact, the harvester used for validation already violates this assumption. One realizes this by comparing the results of Section 5.3 to the curves in Figure 13 because the comparison reveals an apparent contradiction. By Figure 13, at, e.g., τ=40 s, a narrowing of the adjustment bandwidth results in sufficient power for a 2-mW load (black solid curve), but the rule in the bullet list at the end of Section 5.3 asserts the power to be insufficient. The discrepancy is due to the fact that the assumption of constant ${\tilde{W}}_{T}(f)$ disregards the disproportionately high adjustment energies needed when adjusting to the very adjustment bandwidth limits. As a consequence, the new adjustment power $P_{T,B}$ with the ABS factor *s* implemented stays below the value $s^{2}P_{T,A}$ predicted by Equation (10), which in turn results in a bigger net available power $P_{\mathrm{net}}$.

**Figure 14:**
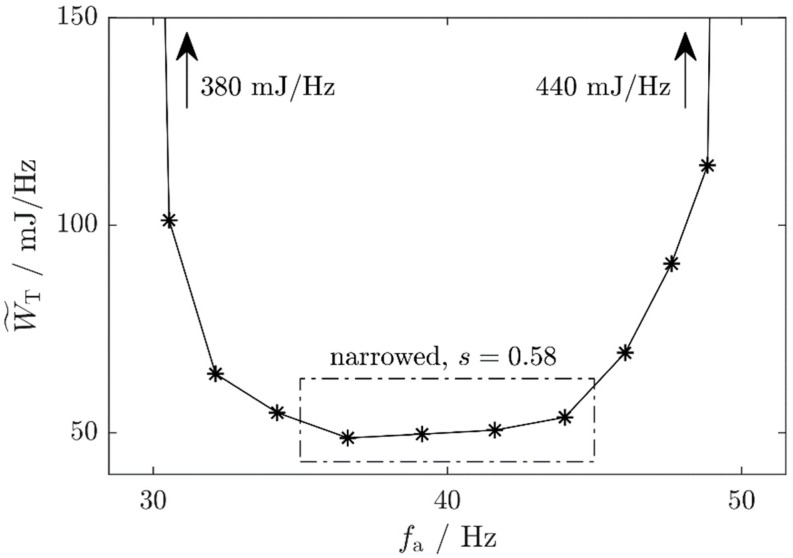
The adjustment energy per frequency interval, ${\tilde{W}}_{T}(f)$, for the harvester used as validation case (computed from data in [25]). The dash-dotted rectangle marks the frequencies to which the adjustment with the ABS factor *s* = 0.58 was allowed.

These are details of the energy harvester’s design. Its resonance frequency depends on the angular position of the cylindrical tuning magnet. When the transition region between the north-pole and south-pole sides of the tuning magnet are nearest to the cantilever magnet, the change of the magnetic flux and therefore the change of the resonance frequency $f_{r}$ per 15° rotation step (i.e., per 124 mJ) is higher than the changes when the tuning magnet poles are nearest to the cantilever magnet [25]. This leads to a significant frequency dependence of ${\tilde{W}}_{T}(f)$ (Figure 14). At the adjustment-range limit, ${\tilde{W}}_{T}$ is much higher, so adjusting to these frequencies should be omitted. The discussed narrowing with s=0.58 only uses the four middle frequencies. Within this bandwidth, ${\tilde{W}}_{T}$ is nearly constant, which justifies the previous assumption of ${\tilde{W}}_{T}$ being constant.

The discussion clearly shows that the optimum mode of operation of an energy harvester depends both on the environment (τ) and the harvester design (${\tilde{W}}_{T}(f)$).

## 6. Summary and Outlook

In this paper, we have examined the performance of self-adapting vibration energy harvesting systems with passive adjustment and have derived design rules, or rather operation rules, that result in an optimized energy output. To the best of the authors’ knowledge, prior work has always treated the adaptivity of an energy harvester as a feature that is either present or absent, but has not investigated if a partial or temporarily discontinued adjustment could outperform a full adaptivity in terms of power output. Our novel rules are based on the insight that a useful self-adaptive system must harvest more energy than the same system with the adaptation algorithm turned off and that the additional power won by the adjustment must exceed the power needed for the adjustment. We were able to show by comparison with a physical energy harvester described in the literature that the harvester output can be improved by partial adaptivity, i.e., by skipping adjustment steps or narrowing the adjustment bandwidth. In the best case, the novel optimum mode of operation increased the power output by a factor of 3.6.

These results have to be evaluated in the context of the validation case considered. The physical harvester considered was subjected to periodic vibration frequency changes. If the environmental changes are not strictly periodic, our design rules only hold in an average sense. At present, no quantitative measure is known that would allow one to state a priori that an instationary environmental process is “approximately periodic enough” to allow our rules to be applied. What can be said with certainty is that a self-adaptive energy harvester does not work optimally in a completely chaotic environment, about which nothing is known because the energy expended on many adjustment steps will be wasted in that it will not be recovered by additionally harvested energy.

Another specific feature of our validation case was the fact that the sequence of vibration frequencies was completely known a priori (time instants of frequency changes, frequency values). In practice, this will hardly be the case. One therefore needs continuous measurements to detect environmental changes and to initiate an adjustment step if necessary. The aim of future research is to collect more information about concrete application scenarios to make more reliable predictions, and to collect this information in an energy-efficient manner.

## Figures and Tables

**Table 1:** Powers (@ τ = 70 s) and minimum process stationarity times τ_0_, τ_1_ for various operating modes of the self-adaptive harvester from Reference [25].

Mode of Operation	*P*_0_/mW	*P*_T_/mW	*P*_net_/mW	τ_0_/s	τ_1_/s	Opt. Solution If
Non-adjusting system	0.85		0.85			τ < 26 s
Adjusting system						
Strictly periodic (Reference [25])	10.1	8.7	1.3	61	76	
This work						
Skip adjustment to *f*_a,8_	10.1	6.2	3.8	44	55	112 s < τ
Skip adjustment to *f*_a,4_ and *f*_a,8_	9.0	4.6	4.4	36	46	79 s < τ < 112 s
Narrowed range, *s* = 0.58	6.9	2.3	4.7	23	33	26 s < τ < 79 s

## Abbreviations

**Symbol**	**Meaning**
$f_{a}$; $f_{a,i}$	ambient vibration frequency; *i*-th ambient vibration frequency
$f_{a,\mathrm{low}}$, $f_{a,\mathrm{high}}$	lowest and highest ambient vibration frequency
$f_{r}$; $f_{r,i}$	resonance frequency of energy harvester; *i*-th resonance frequency
Δf	frequency spacing for possible adaptation step
$\Delta f_{L}$	upper limit of Δf for positive net output energy of harvester
$\bar{\Delta f}$; ${\bar{\Delta f}}_{A}$, ${\bar{\Delta f}}_{B}$	average frequency spacing for periodic adjustment; same before (A) and after (B) adjustment-bandwidth scaling (ABS)
k	ratio between tuning and harvested power before ABS
$k_{\mathrm{eff}}$	effective spring stiffness
m	effective mass
$P_{0}$; $P_{0,A}$, $P_{0,B}$	average harvested power; same before (A) and after (B) ABS
$P_{0m}$	maximum of $P_{0}$
$P_{L}$	load power
$P_{\mathrm{net}}$; $P_{\mathrm{net},A}$, $P_{\mathrm{net},B}$	net available power; same before (A) and after (B) ABS
$\Delta P_{\mathrm{net}}$	gain in net available power of energy harvester
$P_{T}$; $P_{T,A}$, $P_{T,B}$	average tuning power; same before (A) and after (B) ABS
$P_{\mathrm{Tm}}$	maximum tuning power
r	decimation ratio
s	scaling factor for adjustment bandwidth
$s_{1}$, $s_{2}$	limit scaling factors with zero gain in net available power
$s_{\mathrm{opt}}$	optimum scaling factor for maximum gain in net available power
τ	hold phase duration = average time span between adjustment steps = process stationarity time of ambient vibration
$\tau_{0}$	value of τ at which net available power vanishes
$\tau_{1}$	value of τ at which net available power equates load power
$W_{0}$	harvested energy
$W_{T}$	tuning energy
${\tilde{W}}_{T}$	tuning energy per unit frequency

## Appendix A

We wish to calculate the average value of the spacing Δf between the harvester resonance frequencies before and after an adjustment step when the external vibration frequency is a random variable uniformly distributed in a given interval [$f_{a,\mathrm{low}}$, $f_{a,\mathrm{high}}$]. We use the common notation with capitalization for random variables and write $F_{a}$ for the random vibration frequency. Its probability density function (pdf) is $p_{a}(f_{a})=1/(f_{a,\mathrm{high}}-f_{a,\mathrm{low}})$ for $f_{a,\mathrm{low}}<f_{a}<f_{a,\mathrm{high}}$ and else 0. Similar remarks apply to the resonance frequency $F_{r}$ of the energy harvester. Since this $F_{r}$ results from adjustment steps, it obeys the same probability distribution as $F_{a}$. Given the instantaneous resonance frequency $f_{r}$ (a random value in the interval [$f_{a,\mathrm{low}}$, $f_{a,\mathrm{high}}$]) and the instantaneous vibration frequency $f_{a}$ (also a random value in [$f_{a,\mathrm{low}}$, $f_{a,\mathrm{high}}$]), the frequency spacing is $\Delta f=\left|f_{a}-f_{r}\right|$. Its average or expected value is calculated under the assumption that $f_{r}$ and $f_{a}$ before an adjustment step are independent of each other. In this case, the joint pdf $p_{\mathrm{ar}}(f_{a},f_{r})$ is the product of the individual pdfs, and one obtains:

(A1) $$\begin{array}{c} \bar{\Delta f}=E(|F_{a}-F_{r}|)=\int_{f_{r}=f_{a,\mathrm{low}}}^{f_{a,\mathrm{high}}}\int_{f_{a}=f_{a,\mathrm{low}}}^{f_{a,\mathrm{high}}}|f_{a}-f_{r}|p_{\mathrm{ar}}(f_{a},\;f_{r})\;df_{a}\;df_{r}=\\ =\frac{1}{{\left(f_{a,\mathrm{high}}-f_{a,\mathrm{low}}\right)}^{2}}\int_{f_{r}=f_{a,\mathrm{low}}}^{f_{a,\mathrm{high}}}\int_{f_{a}=f_{a,\mathrm{low}}}^{f_{a,\mathrm{high}}}|f_{a}-f_{r}|\;df_{a}\;df_{r}=\frac{1}{3}(f_{a,\mathrm{low}}-f_{a,\mathrm{high}}). \end{array}$$

## References

[1] Werner-Allen G., Ruiz M., Marcillo O., Johnson J., Lees J., Welsh M. (2006). Deploying a wireless sensor network on an active volcano. IEEE Internet Comput..

[2] Yang J., Zhou J., Lv Z., Wei W., Song H. (2015). A Real-time monitoring system of industry carbon monoxide based on wireless sensor networks. Sensors.

[3] Magno M., Polonelli T., Benini L., Popovici E. (2015). A low cost, highly scalable wireless sensor network solution to achieve smart LED light control for green buildings. IEEE J..

[4] Rawat P., Deep Singh K., Chaouchi H., Bonin J.M. (2014). Wireless sensor networks: A survey on recent developments and potential synergies. J. Supercomput..

[5] Matiko J., Brabham N.J., Beeby S.P., Tudor M.J. (2014). Review of the application of energy harvesting in buildings. Meas. Sci. Technol..

[6] Mitcheson P., Yeatman E.M., Rao G.K., Holmes A.S., Green T.C. (2008). Energy harvesting from human and machine motion for wireless electronic devices. Proc. IEEE.

[7] Cepnik C., Lausecker R., Wallrabe U. (2013). Review on electrodynamic energy harvesters—A classification approach. Micromachines.

[8] Guyomar D., Lallart M. (2011). Recent progress in piezoelectric conversion and energy harvesting using nonlinear electronic interfaces and issues in small scale implementation. Micromachines.

[9] Xu Z., Xi Wang X., Shan X., Xie T. (2017). Parametric analysis and experimental verification of a hybrid vibration energy harvester combining piezoelectric and electromagnetic mechanisms. Micromachines.

[10] Zhu D., Tudor M.J., Beeby S.P. (2010). Strategies for increasing the operating frequency range of vibration energy harvesters: A review. Meas. Sci. Technol..

[11] Tang L., Yang Y., Soh C.K. (2010). Toward broadband vibration-based energy harvesting. J. Intelligent Mater. Syst. Struct..

[12] Pellegrini S., Tolou N., Schenk M. (2012). Bistable vibration energy harvesters: A review. J. Intelligent Mater. Syst. Struct..

[13] Gieras J., Oh J.H., Hauzmezan M., Sane H.S. (2007). Electromechanical Energy Harvesting System.

[14] Wu X., Lin J., Kato S., Zhang K., Ren T., Lui L. A frequency adjustable vibration energy harvester. Proceedings of the PowerMEMS.

[15] Leland E., Wright P. (2006). Resonance tuning of piezoelectric vibration energy scavenging generators using compressive axial preload. Smart Mater. Struct..

[16] Hu Y., Hue H., Hu H. (2007). A piezoelectric power harvester with adjustable frequency through axial preloads. Smart Mater. Struct..

[17] Eichhorn C., Goldschmidtboeing F., Woias P. A frequency tunable piezoelectric energy converter based on a cantilever beam. Proceedings of the PowerMEMS.

[18] Peters C., Maurath D., Schock W., Mezge F., Manoli Y. (2009). A closed-loop wide-range tunable mechanical resonator for energy harvesting systems. J. Micromech. Microeng..

[19] Eichhorn C., Tchagsim R., Wilhelm N., Woias P. (2011). A smart and self-sufficient frequency tunable vibration energy harvester. J. Micromech. Microeng..

[20] Challa V., Prasad M.G., Shi Y., Fisher F.T. (2008). A vibration energy harvesting device with bidirectional resonance frequency tunability. Smart Mater. Struct..

[21] Zhu D., Roberts S., Tudor M.J., Beeby S.P. Closed-loop frequency tuning of a vibration-based micro-generator. Proceedings of the PowerMEMS.

[22] Zhu D., Roberts S., Tudor M.J., Beeby S.P. (2010). Design and experimental characterization of a tunable vibration-based electromagnetic micro-generator. Sens. Actuat. A.

[23] Ayala-Garcia I., Mitcheson P.D., Yeatman E.M., Zhu D., Beeby S.P. (2013). Magnetic tuning of a kinetic energy harvester using variable reluctance. Sens. Actuat. A.

[24] Challa V., Prasad M.G., Fisher F.T. (2010). Towards an autonomous self-tuning vibration energy harvesting device for wireless sensor network applications. Smart Mater. Struct..

[25] Hoffmann D., Willmann A., Hehn T., Folkme B., Manoli Y. (2016). A self-adaptive energy harvesting system. Smart Mater. Struct..

[26] Sun W., Jung J., Seok J. (2015). Frequency-tunable electromagnetic energy harvester using magneto-rheological elastomer. J. Intellignt Mater. Syst. Struct..

[27] Zhou Y., Apo D.J., Shashank P. (2013). Dual-phase self-biased magnetoelectric energy harvester. Appl. Phys. Lett..

[28] Roundy S., Zhang Y. (2004). Toward self-tuning adaptive vibration based micro-generators. Proc. SPIE.

[29] Roundy S. (2005). On the effectiveness of vibration-based energy harvesting. J. Intelligent Mater. Syst. Struct..

[30] Beeby S., Torah R.N., Tudor M.J., Glynne-Jones P., O’Donnell T., Saha C.R., Roy S. (2007). A micro electromagnetic generator for vibration energy harvesting. J. Micromech. Microeng..

[31] Cepnik C., Wallrabe U. (2013). Approaches for a fair comparison and benchmarking of electromagnetic vibration energy harvesters. Micromachines.

[32] Hoffmann D., Willmann A., Folkmer B., Manoli Y. (2014). Tunable vibration energy harvester for condition monitoring of maritime gearboxes. J. Phys. Conf. Ser..

[33] Bronstein I.N., Semendyayev K.A., Musiol G., Mühlig H. (2015). Handbook of Mathematics.

[34] Rao S.S. (2011). Mechanical Vibrations.

